# Barriers and enablers to postnatal care utilization in the Oshana region of Namibia: A qualitative study

**DOI:** 10.1016/j.eurox.2025.100385

**Published:** 2025-03-24

**Authors:** Enos Moyo, Perseverance Moyo, Tafadzwa Dzinamarira, Andrew Ross

**Affiliations:** a University of KwaZulu-Natal, College of Health Sciences, School of Nursing & Public Health, Durban, South Africa; b Medical Centre Oshakati, Oshakati, Namibia; c University of Pretoria, Faculty of Health Sciences, School of Health Systems and Public Health, Pretoria, South Africa

**Keywords:** Barriers, Enablers, Postnatal care utilization, Oshana region, Namibia, Qualitative study

## Abstract

**Background:**

Postnatal care (PNC) service utilization remains low in Namibia, including in the Oshana region, with only 20 % of newborn babies accessing them within two days of delivery in 2021, which is much lower than the 69 % of mothers nationwide who utilized PNC services. As low PNC utilization is linked to high maternal and child morbidity and mortality, this study aimed to explore the barriers and enablers of PNC utilization among women in the Oshana region of Namibia.

**Methods:**

A descriptive qualitative design within an explanatory sequential mixed methods design was used**.** 13 female participants were recruited from the Oshana region's public healthcare facilities through purposive sampling with maximum variation. Semi-structured interviews were conducted, and the data was analyzed thematically.

**Results:**

Six themes and 15 subthemes emerged from the barriers, while five themes and 11 subthemes emerged from the enabling factors. Themes related to barriers and enablers included personal, household, community, cultural, health system, and economic factors.

**Conclusion:**

A comprehensive approach is needed to improve PNC utilization. This includes enhancing PNC knowledge, increasing healthcare accessibility, addressing gender norms and cultural beliefs, and improving the quality of PNC services.

**Statement of Significance**

**Problem**

There is limited evidence on the reasons that influence utilization or non-utilization of postnatal care services by women in the Oshana region of Namibia.

**What is Already Known**

About 69 % of postpartum women utilize postnatal care services within the first two days of giving birth in Namibia. Evidence has identified several individual, household, community, cultural, economic, and health system enablers and barriers to postnatal care utilization globally.

**What this Paper Adds**

Themes related to postnatal care utilization enablers and barriers in the context of Namibia were developed from the perspectives of the women. Recommendations were suggested to address these barriers.

## 1 Introduction

Maternal and neonatal mortality remain significant public health challenges globally, with an estimated 287,000 women and girls having died from pregnancy and childbirth-related complications in 2020, translating to a maternal death every two minutes [1]. Of all the maternal deaths globally, approximately 60 % occur during the postpartum period, which is the first 42 days after delivery [2]. Sub-Saharan Africa (SSA) bears a disproportionate burden of approximately 70 % of the global maternal deaths in 2020. While the global maternal mortality ratio (MMR) in 2020 was 223 maternal deaths per 100,000 live births, in SSA was 545 [1]. The lifetime risk of maternal death, defined as the probability that a 15-year-old girl will die during her lifetime from complications related to pregnancy or delivery, was estimated to be 1 in 41 in SSA in 2020, which is approximately 400 times higher than in Australia and New Zealand [1]. Furthermore, around 67 % of all newborn deaths in SSA occur within the neonatal period, which is the first month after birth [2]. According to UNICEF (2024), there were an estimated 17 neonatal deaths per 1000 live births in 2022 globally [3]. Regionally, the neonatal mortality rate (NMR) was highest in SSA, at 27 deaths per 1000 live births, ranging from as low as 11 in South Africa to as high as 39.4 deaths per 1000 live births in South Sudan [3].

Despite the majority of maternal and child deaths occurring in the postnatal period, there remains a low utilization of postnatal care (PNC) services in SSA, with the average rate between 2006 and 2018 being estimated at around 52 % [4]. Numerous variables that impact PNC use in SSA have been found in earlier research, which can be subdivided into aspects related to the individual, community, culture, health system, and economy. Some of the personal factors include the woman's level of education [5], [6], [7], age [8], [9], employment status [10], [11], level of PNC knowledge [12], [13], availability of a domestic helper [14], and their ability to choose whether or not to utilize PNC services [15], [16], while community factors include influence from peers and elderly women [17], and community-based health support [18]. Some examples of health system factors are the distance to a healthcare facility [7], [19], place of delivery [20], [21], attitude of healthcare workers [22], [23], and number of antenatal care (ANC) visits [5], [24]. Economic factors include household income [25], [26], while cultural factors include indigenous beliefs related to the causes of ill health and therefore the treatment modalities [22], [27].

In Namibia, the MMR was estimated to be 215 deaths per 100,000 live births in 2020 [1], while the NMR in 2022 was 18.7 deaths per 1000 live births [3]. PNC services utilization remains low in Namibia, including in the Oshana region in the north, which is inhabited by a largely rural population. Only 20 % of newborn babies received PNC within two days of delivery in 2021, which is considerably less than the 69 % of mothers who received it nationwide [28]. In this regard, there is limited literature on the factors that influence the utilization of PNC services in the Oshana region. This study, which was part of a larger study on the utilization of PNC services in the Oshana region of Namibia, aimed to explore the barriers and enablers of PNC utilization among women in the region. The findings were expected to assist with formulating strategies to improve PNC utilization in the region, which, it was hoped, would result in a reduction in maternal and neonatal deaths.

## 2 Methods

### 2.1 Study setting and period

The study sites were all 18 public healthcare facilities in the Oshana region of Namibia, which is one of 14 regions in the country, with a population of approximately 175,000 people [29]. Public healthcare facilities in the Oshana region include five health centers, 12 primary healthcare clinics, and one hospital [30]. The study was conducted in the Oshana region, as, together with the Khomas region, it contributed to approximately 50 % of the 48 maternal deaths in Namibia in 2017 [31]. Data collection was conducted from selected public health facilities from October 1, 2023, to January 30, 2024.

### 2.2 Study design

The larger study followed an explanatory, sequential, mixed methods design, with this manuscript presenting the results of the qualitative arm, which followed a descriptive, qualitative design to explore the barriers and enablers of PNC utilization among women in the Oshana region of Namibia.

### 2.3 Study population

The study population included all women of reproductive age residing in the Oshana region of Namibia.

### 2.4 Sample size

A systematic review to determine the sample size required for saturation in qualitative studies revealed that 9–17 participants were adequate [32]. Therefore, the provisional sample size for the face-to-face interviews was 18 participants, nine from those utilizing PNC services, and another nine from those utilizing ANC but not PNC services after their last delivery. However, during data collection, data saturation determined the actual size, which was 13, five being those utilizing PNC services after their last delivery, while eight were utilizing ANC but had not utilized PNC services after their last delivery.

### 2.5 Inclusion criteria

Participants who had taken part in the quantitative arm of the larger parent study were requested to participate in face-to-face interviews soon after completing the questionnaire. Participants included in the quantitative phase of the study were women who had given birth in the five years before the study and were attending ANC or PNC at all 18 public healthcare facilities in the Oshana region.

### 2.6 Exclusion criteria

Women who were seriously ill, unable to communicate clearly due to illness, or unwilling to sign consent were excluded from the study.

### 2.7 Sampling method

A purposive, non-random sampling method with maximum variation was used to select the participants who took part in the study and entailed selecting those from the quantitative phase of the study who met the inclusion criteria. The sample included participants with varying socio-demographic characteristics, such as age, marital status, place of residence, highest educational attainment, and employment status. One participant was recruited from each of the 18 healthcare facilities for the interviews, the number included depending on when data saturation was achieved.

### 2.8 Data collection

One of the researchers (EM), who is a public health specialist and was pursuing a doctorate in public health at the time of the study, conducted face-to-face, semi-structured interviews with a translator. EM has extensive experience in conducting qualitative research, as he has been involved in collecting qualitative data in several studies conducted in Namibia for his academic research and for some organizations in the country. While the interviews were conducted in English, where a participant was not sufficiently fluent, the translator translated the questions being asked by the researcher (EM) into Oshikwanyama, and explained the participants’ responses to the researcher. The researcher asked the participants several questions to determine the reasons they did or did not utilize PNC services. A topic guide that was pretested for the interviews was followed, being available in both English and Oshikwanyama, the local language. Forward and back translations of the topic guide were carried out to ensure the accuracy of the Oshikwanyama version. The English version of the topic guide is attached as Appendix 1. The researcher conducted the interviews at the healthcare facilities, which lasted 30–45 minutes, and were audio-recorded for later verbatim transcription and analysis.

### 2.9 Trustworthiness

The components that are used to enhance trustworthiness in qualitative research include credibility, confirmability, dependability, and transferability [33]. To enhance credibility, the researcher gave sufficient time to the participants to respond to all the questions, while the researcher kept field notes and engaged a qualified qualitative researcher to determine whether he agreed with the researcher's coding decisions. Confirmability was addressed by undertaking peer debriefing sessions with the other researchers involved in the study in which the principal researcher (EM) presented oral summaries of the data collected, emerging themes, subthemes, and interpretations of the data. Dependability was enhanced through the use of an audit trail of the research decisions, data analysis, and processes to illustrate the transparency of the decisions that were made in the qualitative inquiry. To address transferability, the researchers provided thick descriptions of the participant’s views, including direct quotes.

### 2.10 Data analysis

Thematic analysis, which involves a rich, detailed yet complex account of the data, was used to analyse the data. It is useful for examining the perspectives of different research participants, highlighting their similar and different perspectives while generating unanticipated insights, and ensuring that the researcher takes a well-structured approach to handling data [34]. The process of thematic analysis involved familiarisation, coding, then generating, reviewing, defining and naming themes, interpreting them, and checking the findings [34]. One of the researchers (EM) transcribed the audio recordings to become familiar with the data, while two (EM and PM), who read and reread each interview transcript while listening to the audio recordings, verified the accuracy of the transcripts. An expert in qualitative research (TM), who was also part of the research team, analyzed the data independently, with consensus discussions being held to discuss the codes, emerging themes, and subthemes from the data as it was collected. NVivo version 20 software was used for the coding process. NVivo 20 is a qualitative data analysis software that facilitates the systematic organization, coding, and analysis of large volumes of text data. It allows researchers to identify patterns, categorize data, and generate nodes (codes) that represent significant themes or concepts. The relationships module in NVivo 20 was used to group the nodes into similar concepts of barriers and enablers of PNC utilization among women.

### 2.11 Ethical considerations

The Biomedical Research Ethics Committee at the University of KwaZulu-Natal (Protocol reference number: BREC/00005788/2023) and Namibia's Ministry of Health and Social Services (Ref: 22/4/2/3) both granted the study ethical clearance. Participation in this study was voluntary and informed consent was obtained from each participant.

## 3 Results

### 3.1 Participants’ characteristics

Of the 18 women who were expected to participate, only 13 were interviewed since data saturation was reached after these interviews. Five were recruited from those attending PNC services, while eight were attending ANC and had not utilized PNC after their last delivery (Table 1). There were three participants each in the age groups 18–25, 31–35 and 36–40 years, two in the age group 26–30 years, and one each in the age groups 41–45 and 46–50 years. The majority of participants were single (n = 7), stayed in urban areas (n = 8), had primary or secondary education (n = 4 each), and were employed (n = 9). Most of the participants (n = 10) had attended ANC during their last pregnancy, while a few (n = 5) had utilized PNC services after their last pregnancy.

**Table 1:** Participants’ characteristics.

**ID**	**Age group**	**Marital status**	**Residence**	**Highest level of education**	**Employment status**	**Attended ANC**	**PNC Utilization**
1	31 – 35	Single	Rural	Secondary	Employed	Yes	Yes
2	46 – 50	Married	Rural	No formal education	Unemployed	Yes	No
3	18 – 25	Single	Urban	Secondary	Employed	Yes	No
4	41 – 45	Married	Urban	Tertiary	Employed	No	Yes
5	36 – 40	Divorced	Urban	Primary	Unemployed	Yes	No
6	36 – 40	Married	Urban	Tertiary	Employed	No	Yes
7	26 – 30	Single	Urban	Secondary	Employed	Yes	No
8	18 – 25	Single	Rural	Tertiary	Employed	Yes	No
9	18 – 25	Single	Urban	Primary	Employed	Yes	Yes
10	36–40	Widowed	Rural	No formal education	Unemployed	Yes	No
11	31 – 35	Divorced	Urban	Primary	Employed	Yes	No
12	26 – 30	Single	Urban	Secondary	Employed	Yes	No
13	31 – 35	Single	Rural	Primary	Unemployed	No	Yes

### 3.2 Themes and sub-themes

Six themes and 15 subthemes emerged from the barriers, while five themes and 11 subthemes emerged from the enabling factors (Table 2).

**Table 2:** Themes and subthemes.

**Theme**	**Subthemes**
**Barriers**	
1. Personal barriers	1. Illness during the postnatal period 2. Lack of PNC knowledge or no need to attend
2. Household barriers	1. Unavailability of person to leave at home 2. Husband’s decision 3. Household activities requiring the attention of women
3. Community barriers	1. Community concerns about PNC 2. Community activities
4. Cultural barriers	1. Protecting newborns from bad spirits 2. Ritual performance
5. Health system barriers	1. Long distance to the clinic 2. Disrespectful nurses 3. Lack of inpatient PNC services 4. Long waiting times
6. Economic barriers	1. Lack of money for transport 2. Need to take part in economic activities
**Enablers**	
1. Personal enablers	1. Postpartum complications among family members 2. Complications during pregnancy
2. Household enablers	1. Availability of people at home 2. Appointment reminders from the husband
3. Community enablers	1. Encouragement by friends and family members 2. Encouragement by neighbours
4. Health system enablers	1. Proximity to the clinic 2. Respectful nurses 3. Reminders by community health workers
5. Economic enablers	1. Free services 2. Availability of transport to the clinic

### 3.3 Themes related to barriers

From the data analysis, six themes and 15 subthemes regarding the barriers women faced in utilizing PNC services in the study area emerged.

#### 3.3.1 Theme 1: personal barriers

Two individual factors that hindered PNC utilization were revealed during the interviews, and the subthemes under this theme were illness during the postnatal period and a lack of PNC knowledge.

##### 3.3.1.1 Sub-theme 1: illness during the postpartum period

Participants indicated that they knew about PNC services and the need to attend them, but were not able to do so as they were sick during that period.

*‘I was not feeling well during the time and was being attended at home by a traditional healer.’ (Participant 3, 18–25 years old, single, urban resident, tertiary education)*

*‘I was not feeling well during the time I was supposed to attend PNC visits.’ (Participant 8, 18–25 years old, single, rural resident, tertiary education)*

##### 3.3.1.2 Sub-theme 2: lack of PNC knowledge or no need to attend

Some participants indicated that they did not know about the PNC services or see the need to attend them if their babies were in good health. One participant had this to say:

*‘I believe that it is not helpful to go to the clinic when myself and my baby are not sick. I think it is a waste of my time. I did not attend when I delivered my older two babies and nothing happened to me or them.’ (Participant 12, 26–30 years old, single, urban resident, tertiary education)*

#### 3.3.2 Theme 2: household barriers

This theme had three subthemes, these being the unavailability of a person to leave at home, the husband’s decision, and the presence of household activities that required their attention.

##### 3.3.2.1 Sub-theme 1: unavailability of a person to leave at home

Participants indicated that it was difficult for them to undertake PNC visits if they did not have anyone to leave at home to look after the other children or the house, the latter being necessary to prevent theft.

*‘I did not have anyone to remain at home with my other 3 little children. The father goes to work every day and I am the only one who takes care of them.’ (Participant 2, 46–50 years old, married, rural resident, no formal education)*

*‘I stay alone with my husband at home. So, if I went to the clinic for PNC, it would mean that I leave the house without anyone looking after it. This might have attracted thieves. Our area has a lot of thieves and break-ins occur during the day when people are out.’ (Participant 3, 18–25 years old, single, urban resident, tertiary education)*

##### 3.3.2.2 Sub-theme 2: husband’s decision

It was revealed during the interviews that some women did not utilize PNC services as their husbands instructed them not to go as they did not see the relevance of these visits. One participant commented on the influence of the husband:

*‘My husband said that there was no point in going to the clinic if we did not have any problems as it would be a waste of money. As he is the only one working and providing for us, I had to listen to him.’ (Participant 2, 46–50 years old, married, rural resident, no formal education)*

##### 3.3.2.3 Sub-theme 3: household activities requiring a woman’s attention

Some participants said that they did not utilize PNC services because they had to attend to other responsibilities around the homestead and that providing food for the family was also a priority.

*‘I am the only one who cultivates the field, so if I go to the clinic, no one will do that. Where will I get food to feed my family?’ (Participant 12, 26–30 years old, single, urban resident, tertiary education)*

#### 3.3.3 Theme 3: community barriers

The two sub-themes of this theme consisted of community concerns about PNC and community activities.

##### 3.3.3.1 Sub-theme 1: community concerns about PNC

Several participants revealed that they did not utilize PNC services after hearing how some women in their communities were required to take blood pressure medication or contraception, the latter being associated with concerns about causing cancer.

*‘Some women in the community said that women who went for PNC were started on BP treatment, which is not good for their health. They said a lot of women are started on BP treatment during these visits. I did not want that to happen to me, so I avoided the clinic after my last delivery.’ (Participant 3, 18–25 years old, single, urban resident, tertiary education)*

*‘My mother-in-law advised me not to go for PNC because she said the nurses would force me to take family planning, which would end up causing cancer.’ (Participant 10, 36–40 years old, widowed, rural resident, no formal education)*

##### 3.3.3.2 Sub-theme 2: community activities

Some participants said that they did not utilize PNC services as they had to attend other important community activities, such as funerals.

*‘When I was supposed to go for my six-week visit there was a funeral of my neighbour’s son, so I did not manage to go.’ (Participant 7, 26–30 years old, single, urban resident, secondary education)*

#### 3.3.4 Theme 4: cultural barriers

Some women did not utilize PNC services due to cultural beliefs that prevented babies from leaving the house for a certain period after delivery. This theme had two subthemes, which were ritual performance and protecting newborns from bad spirits.

##### 3.3.4.1 Sub-theme 1: ritual performance

Some participants reported that they could not utilize PNC services as their babies could not leave the house before certain rituals were performed. One such ritual is the hair-cutting ceremony of the new infant, which is usually performed by a specific family member who may not live close by, resulting in the need to wait until they are available, which could take a few weeks, resulting in them missing PNC appointments.

*‘In our culture, once you bring the baby from the clinic after delivery, the baby cannot go out until the ritual of cutting the hair has been performed. However, the person who is appointed to do this in the family is one. So, in my case, the woman who was supposed to cut the child’s hair stayed far away and did not come until the child was three months old. I failed to attend PNC because I could not leave my baby at home.’ (Participant 10, 36–40 years old, widowed, rural resident, no formal education)*

##### 3.3.4.2 Sub-theme 2: protecting newborns from bad spirits

Several participants did not utilize PNC services due to the cultural practice of newborns not leaving their houses when they were very young, as they could not withstand bad spirits. Such an instruction was usually given by an elder, like a grandmother, the indication being that the postpartum woman was not allowed to attend the clinic.

*‘I was not allowed to take my baby out of the house after delivery because my grandmother said the child was not yet strong enough to be exposed to bad spirits outside. She said that this is what we should do in our culture, and failure to abide by this would lead to the child contracting many illnesses, which may even lead to death.’ (Participant 11, 31–35 years old, divorced, urban resident, primary education)*

#### 3.3.5 Theme 5: health system barriers

This theme had four sub-themes, which were long distances to the clinic, disrespectful nurses, a lack of in-patient PNC services, and long waiting times.

##### 3.3.5.1 Sub-theme 1: long distance to the clinic

Several participants revealed that they did not utilize PNC services as the clinics were located far from where they lived, which is a problem for people who live in rural areas where mobile PNC services are not available.

*‘The clinic is very far from my home. For me to go for PNC, I have to first get money for transport. However, getting money these days is difficult.’ (Participant 2, 46–50 years old, married, rural resident, no formal education)*

##### 3.3.5.2 Sub-theme 2: disrespectful nurses

Hearing about, observing, and experiencing rudeness from nurses were mentioned as reasons why some of the women had not utilized PNC services at their local clinics, despite some attending ANC.

*‘When I attended ANC, the nurses at the clinic were rude. They shouted at some of the patients in front of others. I felt that this was degrading us and I therefore avoided going to the clinic for follow-up. I would rather go to another clinic, not the one near my home.’ (Participant 3, 18–25 years old, single, urban resident, tertiary education)*

*‘The nurses at the clinic are very rude. During my ANC visits, they blamed me for getting pregnant early after the last child in front of the other patients. I felt that they disrespected me, and I did not feel comfortable going there during my postnatal period. The other option was to go to another clinic, but the other clinic is far away, and I was not sure if the nurses there were better than the ones at the nearby clinic.’ (Participant 5, 36–40 years old, divorced, urban resident, primary education)*

*‘I always heard other women say they get shouted at by nurses when they go for check-ups. I therefore decided not to go since I did not want to expose myself to such ill-treatment.’ (Participant 12, 26–30 years old, single, urban resident, secondary education)*

##### 3.3.5.3 Sub-theme 3: lack of inpatient PNC services

Women who were admitted to hospitals during their postnatal period did not receive PNC services, as no arrangements were made for them to receive them while in the ward, despite the staff knowing that they had recently given birth.

*‘I was admitted to a hospital far from where I was supposed to attend PNC. I feel that the health workers at the hospital where I was admitted should have made arrangements for me to be seen by the responsible doctors while I was in hospital. However, no one made such an arrangement. It seemed like they only cared about the condition I was referred for.’ (Participant 8, 18–25 years old, single, rural resident, tertiary education)*

##### 3.3.5.4 Sub-theme 4: long waiting times

Some women reported that they did not utilize PNC services due to the long waiting time at the clinics before being seen, which interfered with or prevented them from executing their other responsibilities.

*‘There are usually long waiting lines at the clinic. As a result, if I have other things to do at home, I would not even think of going there for PNC.’ (Participant 10, 36–40 years old, widowed, rural resident, no formal education)*

#### 3.3.6 Theme 6: economic barriers

The two subthemes under this theme were a lack of money for transport and the need to take part in economic activities.

##### 3.3.6.1 Sub-theme 1: lack of money for transport

Some of the women noted that they did not utilize PNC services due to a lack of money for transport, specifically, those who were unemployed or did not have a partner to provide them with money to travel.

*‘I did not have transport money to go to the clinic.’ (Participant 8, 18–25 years old, single, rural resident, tertiary education)*

##### 3.3.6.2 Sub-theme 2: need to take part in economic activities

The interviews revealed that some women did not utilize the PNC services as they needed to take part in economic activities to provide for their families daily.

*‘I did not attend PNC because no one could sell my products at the market while I was not there. Without income from the market, I would not be able to buy food for my family.’ (Participant 12, 26–30 years old, single, urban resident, secondary education)*

### 3.4 Themes related to enablers

Five themes and eleven sub-themes emerged from the enabling factors, the themes being personal, household, community, health system, and economic enablers.

#### 3.4.1 Theme 1: personal enablers

The two subthemes identified under this theme consisted of health problems experienced by family members during the postnatal period and complications during pregnancy.

##### 3.4.1.1 Sub-theme 1: postpartum complications among family members

Participants who had family members who died or experienced health problems during the postnatal period revealed that they prioritized utilizing PNC services out of fear that the same fate would befall them if they did not look after their health.

*‘I lost a cousin soon after delivery. We did not find out the cause of the death but I was scared that if I was not checked, I would also develop complications and die.’ (Participant 1, 31–35 years old, single, rural resident, secondary education)*

*‘My sister developed fits two weeks after she delivered. I was afraid that this could also happen to me. I therefore made sure that I attended all my PNC visits.’ (Participant 9, 18–25 years old, single, urban resident, primary education)*

##### 3.4.1.2 Sub-theme 2: complications during pregnancy

Some participants reported that developing complications during pregnancy influenced them to utilize PNC services as a way of making sure that their conditions were under control.

*‘I developed diabetes during pregnancy and was told I should attend PNC visits so that the nurses could keep checking my sugar. They explained that the diabetes may disappear after delivery. Therefore, I attended PNC visits to ensure that the nurses monitored my condition.’ (Participant 6, 36–40 years old, married, urban residence, tertiary education)*

#### 3.4.2 Theme 2: household enablers

The availability of people to leave at home and reminders from the husband were the two subthemes under this theme.

##### 3.4.2.1 Sub-theme 1: availability of people at home

Some participants revealed that they were able to utilize PNC services as their husbands, family members or helpers were able to remain at home with their other children or look after the house to prevent theft.

*‘I had a helper who could remain with the child. As a result, it was possible for me to go to the clinic for my PNC without worrying about the baby.’ (Participant 1, 31–35 years old, single, rural resident, secondary education)*

*‘My husband was allowed to remain with the kids by his employer every time I had to attend a visit. This made it easy for me to attend PNC visits.’ (Participant 4, 41–45 years old, married, urban resident, tertiary education)*

*‘My mother always came to stay with the other children whenever I wanted to go to the clinic for my PNC. This was very helpful because, without her, I could have missed some of my visits while taking care of the other children’ (Participant 6, 36–40 years old, married, urban residence, tertiary education)*

##### 3.4.2.2 Sub-theme 2: appointment reminders from the husbands

Some women kept their PNC appointments as their husbands reminded them as the date approached and encouraged them to attend.

*‘Furthermore, my husband always reminded me of my PNC visit days.’ (Participant 6, 36–40 years old, married, urban residence, tertiary education)*

#### 3.4.3 Theme 3: community enablers

The two sub-themes under this theme were encouragement by friends and family members, as well as encouragement by neighbors.

##### 3.4.3.1 Sub-theme 1: encouraged by friends and family members

Participants indicated that they were encouraged to utilize PNC services by friends and family members who had avoided complications after delivery by keeping their PNC appointments.

*‘I was encouraged by my friends and family members to go for PNC since they said it would help avoid complications. One family member told me that had she not attended PNC, she might have developed a stroke since her blood pressure was very high after delivery. The high BP was diagnosed at PNC.’ (Participant 1, 31–35 years old, single, rural resident, secondary education)*

##### 3.4.3.2 Sub-theme 2: encouraged by neighbours

Other participants were encouraged by their neighbours to utilize PNC due to the help they had received in preventing their children from becoming ill.

*‘My neighbour, who had delivered six months before me, encouraged me to attend PNC visits because she said the nurses noticed yellow eyes on her baby when it was starting, which was treated immediately to avoid complications.’ (Participant 4, 41–45 years old, married, urban resident, tertiary education)*

#### 3.4.4 Theme 4: health system enablers

Three sub-themes that emerged in this theme are proximity to the clinic, respectful nurses, and reminders by community health workers.

##### 3.4.4.1 Sub-theme 1: proximity to the clinic

Participants who stayed near clinics revealed that this enabled them to utilize PNC services.

*‘The clinic is near our house, so it was easy for me to attend PNC visits.’ (Participant 13, 31–35 years old, single, rural resident, primary education)*

##### 3.4.4.2 Sub-theme 2. respectful nurses

Participants who had good experiences during their previous maternal healthcare services said that this encouraged them to utilize PNC services after their last delivery.

*‘My previous PNC experience was memorable. The nurses were very friendly and taught me how to take care of the baby and myself after delivery. My experience encouraged me to always make sure that I attended all the PNC visits.’ (Participant 4, 41–45 years old, married, urban resident, tertiary education)*

*‘The nurses at the clinic where I attended my ANC and also delivered were very friendly and supportive. They explained everything I was likely to experience during my pregnancy and after pregnancy. They also gave me the dates for when I was expected to be seen after delivery. Their conduct made me feel confident enough to want to go back for PNC visits.’ (Participant 6, 36–40 years old, married, urban residence, tertiary education)*

##### 3.4.4.3 Sub-theme 3: reminders by community health workers

Some of the women indicated that reminders by their local community health workers about when they needed to visit the clinic for their next PNC check-up were very useful.

*‘Community health workers in my area visited me after delivery and reminded me of the dates I was supposed to go for my follow-up visits and those of the baby.’ (Participant 6, 36–40 years old, married, urban residence, tertiary education)*

#### 3.4.5 Theme 5: economic enablers

The two subthemes under this theme were free services and the availability of transport to the clinic.

##### 3.4.5.1 Sub-theme 1: free services

Participants noted that they were able to utilize PNC services as they are free of charge. The unemployed women or those with a low income, were relieved that they did not have to pay.

*‘PNC is provided free of charge at government clinics, and as a result, I was able to utilize PNC services without worrying about money to pay for the services.’ (Participant 1, 31–35 years old, single, rural resident, secondary education)*

*‘We were not charged anything during the visit. All the medications for me and the baby were provided at no cost.’ (Participant 4, 41–45 years old, married, urban resident, tertiary education)*

##### 3.4.5.2 Sub-theme 2. availability of transport to the clinic

The availability of transport to the clinic enabled them to utilize the PNC services, which is an advantage in urban areas in particular and where a family member had a vehicle.

*‘I had available transport, as my husband always dropped me at the clinic and picked me up after I was done.’ (Participant 6, 36–40 years old, married, urban residence, tertiary education)*

## 4 Discussion

The majority of the participants in this study were below the age of 40 years, were single, and had attained primary or secondary education. This reflects the demographic profile of the women in Namibia [29], making our sample relatively representative of the study population. The study revealed that the majority of the participants who did not utilize PNC services were single, employed, had secondary education or below, and were urban residents. These findings concur with the results of a study conducted in Ethiopia [5], which revealed that women with secondary education or below were unlikely to utilize PNC services, and another study in Rwanda [19], which revealed that employed women were unlikely to utilize PNC services. However, the finding that more urban residents were unlikely to utilize PNC services is at variance with those of a study conducted in Ethiopia [5], which revealed that urban residence was an enabler to PNC utilization. We expected to find more women in urban areas utilizing PNC services due to the proximity of clinics.

### 4.1 Barriers to PNC utilization

This study revealed that personal, household, community, cultural, health system, and economic factors were barriers to PNC utilization. Personal barriers included a lack of PNC knowledge and illness during the postnatal care period, while household barriers included the unavailability of a person to leave at home, a husband's decision, and household activities requiring a woman's attention. Similar to the current study, a study conducted in Ethiopia revealed that a lack of PNC awareness and knowledge hindered women from utilizing PNC services [35]. Women who lack PNC knowledge are less likely to utilize PNC services because they will not know the importance of PNC. The low level of educational attainment among the participants may also have contributed to a lack of PNC knowledge. Although community health workers are tasked with educating women about PNC in the communities in Namibia, to the best of our knowledge, no study has been conducted to assess the effectiveness of this strategy. This study revealed that women who got ill during the postpartum period, who viewed such an illness as needing traditional treatment and did not utilize healthcare services, were unlikely to utilize PNC services. A possible explanation for this finding is that women who do not seek medical treatment when sick may lack trust in the health system, and, therefore, are less inclined to utilize any health services. This study did not delve into illnesses that are considered to be spiritual and may require treatment by traditional healers. Future research may, therefore, be required to have a deeper understanding of illnesses that are considered to be spiritual and their association with the non-utilization of PNC services. The current study revealed that the absence of anyone at home to take care of the other children acted as a barrier to PNC utilization. This finding is consistent with that of a study conducted in Uganda, which revealed that the unavailability of a helper at home was a barrier to PNC utilization [14]. The primary physical caregivers of children in Africa are women [36], which explains these findings. As a result, women need someone to stay with the children when they want to utilize PNC services, especially if their children at home are young. The findings that women's attention-demanding home chores and husbands' decisions serve as obstacles to PNC utilization due to Namibian society's patriarchal and socially imposed gender norms are similar to those of most African societies. As a result, men make most family decisions, such as how household money should be used, and when women should attend maternal healthcare services, while women perform most of the household duties [37].

Community barriers included community concerns about PNC and community activities, while cultural barriers included ritual performance and protection from bad spirits. The community and cultural barriers revealed in the study are consistent with those of a study conducted in Malawi [18]. Community concerns about PNC are usually a result of low health literacy about the postnatal period and PNC services [38]. The cultural barriers identified in this study are similar to those in a study conducted in Ghana, which revealed that babies are confined in the house for a certain period after birth to prevent them from being bewitched [39]. Culture and personal beliefs play a very important role in the health-seeking behavior of Africans. Many of the ethnic groups on the continent believe that illnesses are caused by evil spirits or witchcraft, and usually seek assistance from traditional healers before they go to healthcare institutions [40].

Health system barriers identified included long distances to the clinics, disrespectful nurses, a lack of inpatient PNC services, and long waiting times at the clinics, while economic barriers included a lack of money for transport to the clinic and the need to take part in economic activities. These finding are consistent with a study conducted in Ethiopia which highlighted that long distances to the nearest healthcare facility were a barrier for utilization of PNC services [22]. Long distances to a healthcare facility may prohibit women from utilizing PNC services because they may not have the money for transport, there might be a lack of reliable transport, or there may be poor road networks [41]. Similar to the current study, a study carried out in Namibia revealed that some nurses verbally and physically abused patients, while others just treated them rudely [42]. Numerous issues, including staffing shortages, a lack of managerial support, work overload, and patients' attitudes toward healthcare providers, have been linked to the disrespectful care provided by nurses [43].

### 4.2 Enablers of PNC utilization

This study also identified personal, household, community, health system, and economic enablers to PNC utilization. Personal enablers included postnatal complications among family members and complications during pregnancy, while household enablers included the availability of people to leave at home and reminders from the husbands. A study conducted in Tanzania highlighted that women who experienced complications during pregnancy or delivery were more likely to utilize PNC services [44]. A possible explanation for this finding is that women who experience complications during pregnancy or delivery may need to be reviewed for a certain period after delivery, and this may coincide with their PNC visits. Additionally, patients who lose family members or have family members who develop complications during the postpartum period may be aware of the seriousness of diseases that occur during this period, and, therefore, will be more willing to attend PNC visits.

Community enablers included encouragement from friends, family members, and neighbors, health system enablers included proximity to the clinics, respectful care by nurses, and reminders by community healthcare workers, and economic factors including free services and the availability of transport to the clinic. Community health workers enhance PNC mobilization through community mobilization, patient tracking, and the provision of other services such as family planning [45]. A study conducted in South Africa revealed that mothers appreciated the role performed by community health workers because they understood their life experiences and provided advice and support that were relevant [46]. The finding that free PNC services were an enabler of utilization concurs with the findings of a study conducted in Kenya, which revealed that a free maternal healthcare policy increased the utilization of maternal healthcare services [47]. It should, however, be noted that a free service policy on its own may not increase utilization unless the services are also accessible, easily available, and women-friendly.

### 4.3 Recommendations

We made several recommendations to improve the uptake of PNC services in the Oshana region of Namibia based on the identified barriers and enablers, the latter providing insight into how some issues need to be addressed. PNC information needs to be disseminated to communities through various channels, such as social media, mass media and health promotion activities. Health education and promotion activities conducted by community health workers in Uganda were found to reduce harmful cultural beliefs associated with pregnancy and childbirth, as well as the postpartum period, and improve the knowledge and utilization of ANC and PNC services by women [48]. Community leaders should also be engaged to address gender norms and cultural beliefs that act as barriers to PNC utilization. Governments need to take services to people in rural areas, either through fixed or mobile clinics, since many rural areas are under-serviced. Healthcare workers should be trained in respectful care so that women can be motivated to attend PNC services.

### 4.4 Strengths and limitations of the study

This study provided a broad understanding and insight into factors that influence PNC utilization by women in the Oshana region of Namibia. The use of a descriptive qualitative design enabled participants to indicate the factors affecting the utilization of PNC services. Additionally, the use of a purposive sampling with maximum variation enabled the selection of a sample based on a variety of participants’ characteristics. However, as participants were recruited from healthcare facilities, those who did not utilize them for either ANC or PNC services were not included, possibly introducing selection bias.

## 5 Conclusion

This study revealed several themes that identified the barriers and enablers to PNC utilization in the Oshana region of Namibia. A multi-pronged approach that addresses PNC knowledge, increases access to healthcare services, addresses gender norms and cultural beliefs that hinder PNC utilization, and improves the quality of PNC services is required.

## Funding

This study was not funded.

## CRediT authorship contribution statement

**Moyo Enos:** Writing – original draft, Investigation, Formal analysis, Conceptualization. **Moyo Perseverance:** Writing – original draft, Investigation. **Ross Andrew:** Writing – review & editing, Supervision. **Dzinamarira Tafadzwa:** Writing – review & editing, Supervision.

## Declaration of Competing Interest

The authors declare that they have no known competing financial interests or personal relationships that could have appeared to influence the work reported in this paper.

## Figures and Tables

**Table:** 

**Topic**	**Questions**
1. Antenatal care utilisation	1. Did you attend ANC during your last pregnancy?
2. PNC information	1. If you attended ANC, were you informed about PNC services? If so, what were you told?
3. Reasons for utilising/not utilising PNC services	1. Are there personal reasons why you did/did not attend PNC? If yes, what are the personal reasons? 2. Are there household factors that allowed/prevented you from attending PNC? What are these household factors? 3. Are there community factors that allowed/prevented you from attending PNC? What are these community factors? 4. Are there cultural reasons that allowed/prevented you from attending PNC? What are these cultural factors? 5. Are there health system factors that allowed/prevented you from attending PNC? What are these health system factors? 6. Are there economic factors that allowed/prevented you from attending PNC? What are these economic factors? 7. Are there any other factors that allowed/prevented you from attending PNC? What are these factors?

## Data Availability

The data associated with this manuscript can be provided on request from the authors.

## References

[1] WHO UNICEF, UNFPA, World Bank Group, and UNDESA/Population Division. Trends in maternal mortality 2000 to 2020: estimates from WHO, UNICEF, UNFPA, World Bank Group, and UNDESA/Population Division. 2023.Available at: 〈https://www.who.int/publications/i/item/9789240068759〉 (Accessed: 5 April 2024).

[2] Beraki G.G., Tesfamariam E.H., Gebremichael A., Yohannes B., Haile K., Tewelde S. (2020). Knowledge on postnatal care among postpartum mothers during discharge in maternity hospitals in Asmara: a cross-sectional study. BMC Pregnancy Childbirth.

[3] United Nations International Children’s Emergency Fund (UNICEF). Neonatal mortality. 2021. Available at: 〈https://data.unicef.org/topic/child-survival/neonatal-mortality/〉(Accessed: 21 April 2024).

[4] Tessema Z., Yazachew L., Tesema G., Teshale A. (2020). Determinants of postnatal care utilization in sub-Saharan Africa: a meta and multilevel analysis of data from 36 sub-Saharan countries. Ital J Pedia.

[5] Berhe A., Bayray A., Berhe Y., Teklu A., Desta A., Araya T. (2019). Determinants of postnatal care utilization in Tigray, Northern Ethiopia: a community based cross-sectional study. PLoS ONE.

[6] Dona A., Tulicha T., Arsicha A., Dabaro D. (2022). Factors influencing utilization of early postnatal care services among postpartum women in Yirgalem town, Sidama Regional State, Ethiopia. SAGE Open Med.

[7] Golla B., Berihun S., Taddele M., Abebe Y. (2018). Factors affecting to have postnatal care service on reproductive age group women who had live birth in the recent last two years, Womberma, Woreda, West Gojjam Zone, North, West Ethiopia. Food Nutr J.

[8] Chemir F., Gelan M., Sinaga M. (2018). Postnatal care service utilization and associated factors among mothers who delivered in shebe sombo woreda, Jimma Zone, Ethiopia. Int J Women’s Health Wellness.

[9] Olajubu A. (2021). Non-utilization of Postnatal Care Services and Related Factors among Women in Osun State, Nigeria. J Midwifery Reprod Health.

[10] Khaki J., Sithole L. (2019). Factors associated with the utilization of postnatal care services among Malawian women. Malawi Med J.

[11] Shibru A., Belihu A., Abdissa G. (2018). Postnatal care services utilization and its associated factors among women who gave birth in the past one year in Gulele Sub City, Addis Ababa, Ethiopia. J Health Med Nurs.

[12] Akibu M., Tsegaye W., Megersa T., Nurgi S. (2018). Prevalence and determinants of complete postnatal care service utilization in Northern Shoa, Ethiopia. J Pregnancy.

[13] Sagawa J., Kabagenyi A., Turyasingura G., Mwale S. (2021). Determinants of postnatal care service utilization among mothers of Mangochi district, Malawi: a community-based cross-sectional study. BMC Pregnancy Childbirth.

[14] Kyabaishiki A., Omona K. (2021). Factors Influencing utilization of postnatal care services among postnatal mothers who delivered from China-Uganda friendship hospital, Kampala District. Health Policy Dev.

[15] Chungu C., Makasa M., Chola M., Jacobs C. (2018). Place of delivery associated with postnatal care utilization among childbearing women in Zambia. Front Public Health.

[16] Saol T., Argaw Z., Facha W. (2021). Postnatal care utilization and associated factors among mothers who delivered in the last twelve months in Sodo Zuria District of Wolaita Zone; Southern Ethiopia: a community-based cross-sectional study. Prim Health Care: Open Access.

[17] Tesfaye G., Chojenta C., Smith R., Loxton D. (2019). Magnitude and correlates of postnatal care utilization among reproductive aged women in a rural district in eastern Ethiopia: a cross-sectional study. Midwifery.

[18] Nyondo-Mipando A.L., Chirwa M., Kumitawa A., Salimu S., Chinkonde J., Chimuna T.J. (2023). Uptake of, barriers and enablers to the utilization of postnatal care services in Thyolo, Malawi. BMC Pregnancy Childbirth.

[19] Kawuki J., Gatasi G., Sserwanja Q. (2022). Prevalence of adequate postnatal care and associated factors in Rwanda: evidence from the Rwanda demographic health survey 2020. Arch Public Health.

[20] Alemu T., Demena M., Assebe T., Rad M., Erchafo B. (2021). Early postnatal care utilization and associated factors among mothers who gave birth in the last twelve months in Lemmo District, Hadiya Zone, Southern Ethiopia. Prim Health Care: Open Access.

[21] Dey T., Ononge S., Weeks A., Benova L. (2021). Immediate postnatal care following childbirth in Ugandan health facilities: an analysis of demographic and health surveys between 2001 and 2016. BMJ Glob Health.

[22] Amsalu G., Talie A., Gezimu W., Duguma A. (2022). Non-utilization of postnatal care and its associated factors among women who gave birth in rural districts of Northern Ethiopia: a community-based mixed-method study. Women’s Health (Lond).

[23] Roméo P.S., Badirou A., Véronique T.M., Georgia D., Paul A. (2018). Poor use of postnatal care service at health facilities in rural Southern Benin: What factors should we target?. J Community Health Manag.

[24] Gebre G., Kote M., Tunje A. (2019). Early postnatal care service utilization and sssociated factors among mothers who gave birth in the last one year preceding the survey in Sidama Zone Malga District, Southern Ethiopia. Ethiop J Reprod Health.

[25] Gebreslassie G.T., Mekonen H.H., Hailu G.T., Kiros K.G., Gebreslassie B., Teklu G. (2020). Prevalence and associated factors of early postnatal care service use among mothers who had given birth within the last 12 months in Adigrat Town, Tigray, Northern Ethiopia, 2018. Int J Women'S Health.

[26] Mamuye S. (2020). Magnitude and determinants of postnatal care service utilization among women who gave birth in the last 12 months in Northern Ethiopia: a cross-sectional study. Int J Women'S Health.

[27] Balde M.D., Diallo A., Soumah A.M., Sall A.O., Diallo B.A., Barry F. (2021). Barriers to utilization of postnatal care: a qualitative study in Guinea. Open J Obstet Gynecol.

[28] United Nations International Children’s Emergency Fund (UNICEF). Namibia key demographic indicators. UNICEF; n.d. Available at: 〈https://data.unicef.org/country/nam/〉(Accessed: 9 April 2024).

[29] Namibia Statistics Agency (NSA) (2016).

[30] Ministry of Health and Social Services (MOHSS). Namibia Master Health Facility List. MOHSS; 2018. Available at: 〈https://mfl.mhss.gov.na/location-manager/locations〉 (Accessed: 9 June 2023).

[31] Namibia Statistics Agency (NSA) (2020).

[32] Hennink M., Kaiser B. (2022). Sample sizes for saturation in qualitative research: a systematic review of empirical tests. Soc Sci Med.

[33] Ahmed S. (2024). The pillars of trustworthiness in qualitative research. J Med Surg Public Health.

[34] Polit D., Beck C. (2017). NURSING RESEARCH: Generating and Assessing Evidence for Nursing Practice.

[35] Tesfahun F., Worku W., Mazengiya F., Kifle M. (2014). Knowledge, Perception and utilization of postnatal care of mothers in Gondar Zuria District, Ethiopia: a cross-sectional study. Matern Child Health J.

[36] Hatch M., Posel D. (2018). Who cares for children? A quantitative study of childcare in South Africa. Dev South Afr.

[37] Danforth E.J., Kruk M.E., Rockers P.C., Mbaruku G., Galea S. (2009). Household decision-making about delivery in health facilities: evidence from Tanzania. J Health Popul Nutr.

[38] Probandari A., Arcita A., Kothijah K., Pamungkasari E. (2017). Barriers to utilization of postnatal care at village level in Klaten district, central Java Province, Indonesia. BMC Health Serv Res.

[39] Ansong J., Asampong E., Adongo P.B. (2022). Socio-cultural beliefs and practices during pregnancy, child birth, and postnatal period: a qualitative study in Southern Ghana. Cogent Public Health.

[40] Chukwuneke F., Ezeonu C., Onyire B., Ezeonu P. (2012). Culture and biomedical care in Africa: the influence of culture on biomedical care in a traditional African society, Nigeria, West Africa. Niger J Med.

[41] Sacks E., Finlayson K., Brizuela V., Crossland N., Ziegler D., Sauvé C. (2022). Factors that influence uptake of routine postnatal care: findings on women’s perspectives from a qualitative evidence synthesis. PLoS One.

[42] Wesson J., Hamunime N., Viadro C., Carlough M., Katjiuanjo P., McQuide P. (2018). Provider and client perspectives on maternity care in Namibia: results from two cross-sectional studies. BMC Pregnancy Childbirth.

[43] Haskins J., Phakathi S., Grant M., Horwood C. (2014). Attitudes of nurses towards patient care at a rural district hospital in the KwaZulu-Natal Province of South Africa. Afr J Nurs Midwifery.

[44] Konje E.T., Hatfield J., Sauve R., Kuhn S., Magoma M., Dewey D. (2021). Late initiation and low utilization of postnatal care services among women in the rural setting in Northwest Tanzania: a community-based study using a mixed method approach. BMC Health Serv Res.

[45] Olaniran A., Madaj B., Bar-Zev S., van den Broek N. (2019). The roles of community health workers who provide maternal and newborn health services: case studies from Africa and Asia. BMJ Glob Health.

[46] Wilford A., Phakathi S., Haskins L., Jama N.A., Mntambo N., Horwood C. (2018). Exploring the care provided to mothers and children by community health workers in South Africa: missed opportunities to provide comprehensive care. BMC Public Health.

[47] Masaba B., Mmusi-Phetoe R. (2020). Free maternal health care policy in Kenya; level of utilization and barriers. Int J Afr Nurs Sci.

[48] Okuga M., Kemigisa M., Namutamba S., Namazzi G., Waiswa P. (2015). Engaging community health workers in maternal and newborn care in eastern Uganda. Glob Health Action.

